# Emotional states of different obesity phenotypes: a sex-specific study in a west-Asian population

**DOI:** 10.1186/s12888-021-03131-3

**Published:** 2021-03-04

**Authors:** Fahimeh Mehrabi, Parisa Amiri, Leila Cheraghi, Ali Kheradmand, Farhad Hosseinpanah, Fereidoun Azizi

**Affiliations:** 1 Research Center for Social Determinants of Health, Research Institute for Endocrine Sciences, Shahid Beheshti University of Medical Sciences, P.O.Box: 19395-4763, Tehran, Iran; 2 Department of Epidemiology and Biostatistics, Research Institute for Endocrine Sciences, Shahid Beheshti University of Medical Sciences, Tehran, Iran; 3 Department of Psychiatry, Taleghani Hospital Research Development Committee, Shahid Beheshti University of Medical Sciences, Tehran, Iran; 4 Obesity Research Center, Research Institute for Endocrine Sciences, Shahid Beheshti University of Medical Sciences, Tehran, Iran; 5 Endocrine Research Center, Research Institute for Endocrine Sciences, Shahid Beheshti University of Medical Sciences, Tehran, Iran

**Keywords:** Metabolic syndrome, Obesity, Depression, Anxiety, Stress, Metabolically healthy obesity

## Abstract

**Background:**

The present study aimed to investigate the associations of obesity phenotypes with depression, anxiety, and stress symptoms among adults in the Tehran Lipid and Glucose Study (TLGS).

**Methods:**

Depression, anxiety, and stress levels of participants from the TLGS were examined among different obesity phenotypes in this cross-sectional study.

Obesity was defined as body mass index (BMI) ≥30 kg/m^2^, and metabolically unhealthy status based on having metabolic syndrome (MetS) or type 2 diabetes. Four obesity phenotypes were defined: 1) Metabolically Healthy Non-Obese (MHNO), 2) Metabolically Healthy Obese (MHO) 3) Metabolically Unhealthy Non-Obese (MUNO), and 4) Metabolically Unhealthy Obese (MUO). Emotional states of different obesity phenotypes were assessed by the Persian version of depression, anxiety, and stress scale-21 items (DASS-21). Ordinal logistic regression analysis was used to compare sex-specific odds ratios of depression, anxiety, and stress in different obesity phenotypes.

**Results:**

The mean age of 2469 men and women was 46.2 ± 15.9 and 45.6 ± 14.7, respectively. In total, women were more likely to experience higher levels of depression (30.5%), anxiety (44.2%), and stress (43.5%) symptoms compared to men. After adjusting for potential confounders, compared to MHNO men, the odds of experiencing higher anxiety levels were significantly greater in metabolically unhealthy men whether they were obese (OR: 1.78, 95% CI: 1.25–2.54; *P* = < 0.001) or non-obese (OR: 1.61, 95% CI: 1.17–2.21; *P* = < 0.001), and also in MUO women (OR: 1.73, 95% CI: 1.28–2.34; *P* = < 0.001) compared to MHNO women. Moreover, the odds of experiencing higher stress levels were significantly greater in MUNO men (OR: 1.40, 95% CI: 1.02–1.90; *P* = 0.04) compared to MHNO men and in MUO women (OR: 1.45, 95% CI: 1.07–1.96; *P* = 0.02) compared to MHNO women. No difference in depression levels was observed in either sex.

**Conclusions:**

Our results showed that men and women with various obesity phenotypes experienced different anxiety and stress levels. While MUO women and all metabolically unhealthy men experienced more anxiety and stress levels than MHNO individuals, none of the obesity phenotypes were associated with depression. These findings provide insight into recognizing the psychological consequences of different phenotypes of obesity in both sexes and utilizing future health promotion planning.

**Supplementary Information:**

The online version contains supplementary material available at 10.1186/s12888-021-03131-3.

## Background

Obesity is a common health issue that has tripled over the last decades [1]. Along with the global ascending rate of excessive weight gain, a range of 18.5–25% as an average prevalence for obesity among the Iranian adult population has also been reported [2]. Obesity has been identified as the leading cause of chronic disorders, such as cardiovascular diseases (CVDs), diabetes mellitus, cancers, and premature death [3]. One of the complications of obesity is related to its frequent incidence with metabolic syndrome (MetS). According to some studies, measuring only body mass index (BMI) in evaluating the outcomes of obesity is misleading since individuals’ metabolic status is an equally essential determinant as BMI [4]. In this regard, obesity phenotypes can be represented as combinations of BMI and metabolic health components, leading to different health outcomes [5].

There are fewer investigations about the mental health outcomes of obesity and MetS than physical comorbidities. In the realm of psychological consequences, it has been indicated that metabolic disturbances are partly responsible for increased mortality in schizophrenic and bipolar patients [6]. Yet, there is no certain consensus about the predisposing impacts of obesity and MetS on common mental health outcomes such as depression, anxiety, and stress symptoms. These conditions, which are more common in women than men [7], affect individuals’ moods or feelings, decline productivity, and cause a tremendous economic burden [8, 9]. The prevalence of depression (44%), anxiety (42%), and stress (40%) symptoms among the general population in Iran is surprisingly high, with a higher rate of incidence in women [10]. Due to the sex differences in depression, anxiety, and stress symptoms, in addition to specific physical and socio-environmental determinants in men and women, it seems essential to conduct a sex-specific study to recognize factors affecting them, such as different obesity phenotypes.

Although Individuals with obesity are believed to have more depression and anxiety at clinical and subclinical levels, supported by a large body of evidence [11, 12], some studies have not found any relationship between obesity and mental health outcomes [13–15]. Even others have established hypothesis emphasizing that higher BMI could lead to fewer mental health issues, including depression and anxiety in different populations [16–18]. In terms of MetS, similar conflicting results have been published on depression and anxiety symptoms associated with unfavorable metabolic profiles [19–22]. The previous studies also examined the bi-directional relationship and the existence of a vicious cycle between obesity and stress [23], which has been considered a risk factor for some metabolic syndrome parameters [24]. However, a recent meta-analysis on four qualified studies indicated no relationship between MetS and stress levels in the adult population [24].

The remarkable point is that despite the frequent concurrence of obesity and MetS, most of the prior studies have not considered the heterogeneity of obesity phenotypes. Only a few studies were conducted to assess whether or not being a metabolically healthy obese (MHO) phenotype is a psychologically benign situation compared to other phenotypes, and most of them only considered depression as an outcome [25–29]. Examining the effects of two common somatic diseases on mental health conditions could shed light on one aspect of these multifactorial disorders. The current study aimed to investigate the association of obesity phenotypes with depression, anxiety, and stress symptoms in adult participants of the Tehran Lipid and Glucose Study (TLGS). The obtained results could provide a comprehensive view regarding the emotional states of a large general population in West Asia.

## Methods

### Study design and participants

This study was conducted in the framework of the TLGS. The TLGS includes two major junctures: The first juncture was the 1st phase, a cross-sectional study designed to determine the prevalence of non-communicable diseases (NCDs) risk factors executed from 1999 to 2001. A total of 15,005 individuals aged ≥three who were residents of district 13 of Tehran were recruited in the study. The second juncture includes five follow-up phases that have been implemented from 2002 to 2019 every 3 years. More study details have been reported previously [30].

In the current study, from all the individuals who participated in the TLGS during the 2016–2019 (6th phase), 2728 participants aged ≥20 years with complete data on depression, anxiety, and stress were recruited. After excluding those with missing data on BMI, MetS components, or covariates (sociodemographic factors, smoking status, and level of physical activity) (*n* = 259), the final data of 2469 adults (1158 men and 1311 women) were analyzed. This study was approved by the ethics committee of the Research Institute for Endocrine Sciences, Shahid Beheshti University of Medical Sciences. All the participants signed the written informed consent before data collection.

### Definition and measurements

#### Biochemical measurements

The blood sample was taken from the participants after a 12–14 h overnight fast by trained personnel in the data collection center of the TLGS. All blood samples of fasting blood sugar (FBS) and serum lipids (total cholesterol (TC), high-density lipoprotein cholesterol (HDL-C), and triglycerides (TG)) were analyzed in the TLGS research laboratory on the same day. Additional information has been previously published about the biochemical [31].

#### Clinical and anthropometric measurements

Trained personnel measured the weight, height, and waist circumferences of participants while they wore light clothes and were barefoot. The weight was measured using an electronic digital scale that its accuracy was up to 100 g. The height was examined in cm, while participants were standing normally using a tape meter stadiometer. Waist circumference was measured via an unstretched measuring tape and recorded to the nearest 0.1 cm. The blood pressure was measured after a 15-min rest period in the seated position twice by qualified physicians via a standard mercury sphygmomanometer; the average of two measurements was considered for analysis.

#### MetS and type 2 diabetes

MetS was defined as having any three of the following abnormalities: 1) ethnic-based abdominal obesity, which was defined as waist circumference ≥ 90 cm for men and women [32]; 2) HDL-cholesterol < 40 mg/dl in men or 50 mg/dl in women; 3) triglyceride ≥150 mg/dl; 4) glucose FBS ≥ 126 mg/dl or known treatment for diabetes; 5) blood pressure ≥ 130/85 mmHg or use of antihypertensive drugs [33]. Type 2 diabetes was defined as fasting blood sugar FBS ≥ 126 mg/dl or 2-h post-load glucose ≥200 mg/dl or taking medication for diagnosed diabetes. Obesity was defined as BMI ≥ 30 kg/m^2^.

#### Obesity phenotypes

The participants were categorized into four obesity phenotypes: 1) Metabolically healthy non-obese (MHNO), 2) Metabolically healthy obese (MHO), 3) Metabolically unhealthy non-obese (MUNO), and 4) Metabolically unhealthy obese (MUO). Metabolically unhealthy status was defined as having MetS or diabetes type 2, according to the Joint Interim Statement (JIS) and the American Diabetes Association (ADA), respectively.

#### Sociodemographic characteristics

The participants’ age, sex, marital status, job status, and educational level were assessed via a pretest questionnaire. Participants’ educational level was defined as 1) Primary: including people with less than a high school diploma; 2) Secondary: including people with a high school diploma; and 3) Higher: including people with a college degree or higher.

#### Smoking and physical activity status

The smoking habit of participants was classified into two groups: 1) smokers (daily and occasionally smokers) and 2) non-smokers (ex-smokers or never smokers). Physical activity levels were evaluated by the validated Iranian version of the Modifiable Activity Questionnaire (MAQ). The frequency and duration of each leisure-time and work physical activity (standing, housework, and work activities more intense than standing) were calculated as hour/week. Then they were multiplied by the weight and the metabolic equivalent task (MET) of the particular act to calculate the energy expenditure for each domain. Total physical activity was calculated by adding each domain’s energy expenditure and was categorized into three groups of low (< 600), moderate (600–3000), and high (≥3000) physical activity [34].

#### Depression, anxiety, and stress

The Persian version of depression, anxiety, and stress scale-21 items (DASS-21) was used to assess emotional distress among participants. The psychometric properties of the Persian version of DASS-21 have been previously studied among the Iranian population, and its reliability and validity were approved [35]. DASS-21 is a self-report questionnaire, including three scales, and each scale was composed of seven items divided into subscales with similar content. Examples of items in each subscale are “I couldn’t seem to experience any positive feeling at all” for depression; “I experienced trembling (e.g., in the hands)” for anxiety; and “I felt that I was using a lot of nervous energy” for stress. The participants completed this questionnaire by rating each item to reflect their emotional experiences over the past week from 0 (did not apply to me at all) to 3 (applied to me very much). Depression, anxiety, and stress were treated as ordinal variables in the current study. The cut-off scores for conventional severity labels were used as follows: 1) Depression: normal: 0–9, mild: 10–13, moderate: 14–20, and severe: + 21; 2) Anxiety: normal: 0–7, mild: 8–9, moderate: 10–14, and severe: + 15; 3) Stress: normal: 0–14, mild: 15–18, moderate: 19–25, and severe: + 26. According to the DASS-21 scoring structure, each scale in this questionnaire was multiplied by two, so the highest score for each scale was 42 [36].

### Statistical analysis

Continuous variables were expressed as mean ± standard deviation (SD), and categorical variables were expressed as frequency (percentage). The continuous and categorical variables among different obesity phenotypes were compared via the one-way ANOVA and Chi-square test, respectively. To show an overview and a general comparison, mean scores of depression, anxiety, and stress were compared across different obesity phenotypes via the analysis of covariance and age, marital status, education, job status, smoking status, and level of physical activity were considered as adjustments. Ordinal logistic regression was used to explore the associations across obesity phenotypes and ordinal outcomes of depression, anxiety, and stress. Sex-specific odds ratios (ORs) with 95% confidence intervals were calculated and reported for men and women separately; model 1 was unadjusted, while model 2 was adjusted for age, marital status (Ref.: Married), education (Ref.: Higher), job status (Ref.: Employed), smoking status (Ref.: Non-smoker), and level of physical activity (Ref: High). All tests were two-sided, and a *p*-value of less than 0.05 was considered statistically significant. After controlling the false discovery rate (FDR = 0.2) by the method of Benjamini-Hochberg [37], all *p*-values under 0.04 remained significant. Statistical analysis was conducted using the IBM SPSS 24 (SPSS Inc., Chicago, IL, USA).

## Results

The mean age of 2469 men and women was 46.2 ± 15.9 and 45.6 ± 14.7 years, respectively. The distribution of sociodemographic factors, smoking status, and level of physical activity among study groups are illustrated in Table 1. The prevalence of obesity phenotypes is 45.9% (MHNO) group (45.9%), 23.6% (MUNO), 20.9% (MUO), 9.6% (MHO). The majority of participants in all groups were married (79.9% men and 75.1% women). Most metabolically unhealthy male subjects had a college degree (40% non-obese and 43.4% obese), while most metabolically unhealthy women had a high school diploma or less (45.6% non-obese and 51.1% obese). Most men across all phenotypes (74%) were employed, while most women (71.5%) were unemployed or identified as housewives. The prevalence of type 2 diabetes among MUNO and MUO phenotypes was 20 and 16.6% among men, likewise 33.8 and 25.1% among women. The mean BMI of participants was 27.79 ± 4.85. The descriptive statistics of BMI and MetS components in men and women are represented in Table 1-Additional file 1.

**Table 1:** Distribution of participants‘characteristics in different obesity phenotypes (*n* = 2469)

Variables		Total	Metabolically healthy		Metabolically unhealthy		***P***-value
			Non-obese ***n*** = 1133 (45.9)	Obese ***n*** = 237 (9.6)	Non-obese ***n*** = 583 (23.6)	Obese ***n*** = 516 (20.9)	
**Men**	**Age (year)**	46.2 ± 15.9	41.36 ± 15.8	48.5 ± 12.9	54.1 ± 15.2	40.9 ± 11.6	< 0.001
	**Marital status n(%)**						< 0.001
	Single	233(20.1)	168(32.8)	16(18.6)	29(8.2)	20(9.8)	
	Married	925(79.9)	344(67.2)	70(81.4)	326(91.8)	185 (90.2)	
	**Level of education n(%)**						< 0.001
	Primary	219 (18.9)	62 (12.1)	14 (16.3)	101 (28.5)	42 (20.5)	
	Secondary	481 (41.5)	216 (42.2)	34 (39.5)	142 (40.0)	89 (43.4)	
	Higher	458 (39.6)	234 (45.7)	38 (44.2)	112 (31.5)	74 (36.1)	
	**Job status n(%)**						< 0.001
	Unemployed, but had other sources of income	212 (18.3)	59 (11.5)	4 (4.7)	118 (33.2)	31 (15.1.8)	
	Unemployed/housewife	89 (7.7)	65 (12.7)	4 (4.7)	13 (3.7)	7 (3.4)	
	Employed	857 (74.0)	388 (75.8)	78 (90.7)	224 (63.1)	167 (81.5)	
	**Smoking status n(%)**						0.21
	Smoker	498 (43.0)	213 (41.6)	33 (38.4)	151 (42.5)	101 (49.3)	
	Non-smoker	660 (57.0)	299 (58.4)	53 (61.6)	204 (57.5)	104 (50.7)	
	**Level of Physical activity n(%)**						0.02
	Low	451 (38.9)	174 (34.0)	36 (41.9)	150 (42.3)	91 (44.4)	
	Moderate or High	707 (61.1)	338 (66.0)	50 (58.1)	205 (57.7)	114 (55.6)	
**Women**	**Age (year)**	45.6 ± 14.7	38.3 ± 12.3	46.2 ± 12.2	55.5 ± 13.2	48.5 ± 12.9	< 0.001
	**Marital status n(%)**						< 0.001
	Single	184 (14.0)	148 (23.8)	13 (8.6)	12 (5.3)	11 (3.5)	
	Divorced/Widowed	142 (10.8)	28 (4.5)	17 (11.3)	39 (17.1)	58 (18.6)	
	Married	985 (75.1)	445 (71.7)	121 (80.1)	177 (77.6)	242 (77.8)	
	**Level of education n(%)**						< 0.001
	Primary	372 (28.4)	59 (9.5)	50 (33.1)	104 (45.6)	159 (51.1)	
	Secondary	525 (40.0)	273 (44.0)	60 (39.7)	84 (36.8)	108 (34.7)	
	Higher	414 (31.6)	289 (46.5)	41 (27.2)	40 (17.5)	44 (14.1)	
	**Job status n(%)**						< 0.001
	Unemployed, but had other sources of income	132 (10.1)	26 (4.2)	12 (7.9)	42 (18.4)	52 (16.7)	
	Unemployed/housewife	937 (71.5)	431 (69.4)	112 (74.2)	153 (67.1)	241 (77.5)	
	Employed	242 (18.5)	164 (26.4)	27 (17.9)	33 (14.5)	18 (5.8)	
	**Smoking status n(%)**						0.38
	Smoker	69 (5.3)	38 (6.1)	4 (2.6)	11 (4.8)	16 (5.1)	
	Non-smoker	1242 (94.7)	583 (93.9)	147 (97.4)	217 (95.2)	295 (94.9)	
	**Level of Physical activity n(%)**						0.71
	Low	383 (29.2)	173 (27.9)	44 (29.1)	68 (29.8)	98 (31.5)	
	Moderate or High	928 (70.8)	448 (72.1)	107 (70.9)	160 (70.2)	213 (68.5)	

Data are presented as mean ± SD and frequency (%). Variables were compared via the one-way ANOVA and Chi-square test

**Fig. 1:**
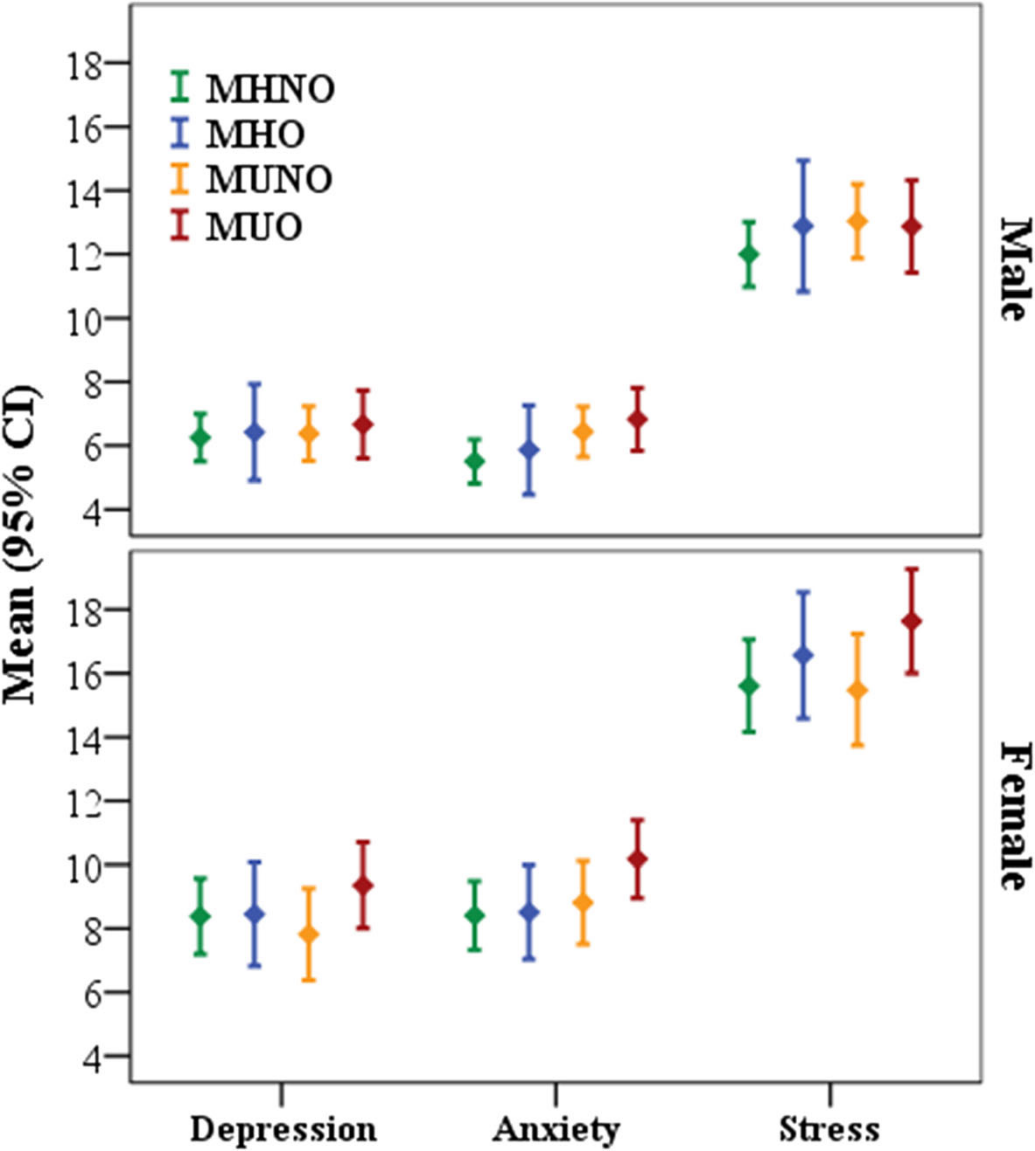
The depression, anxiety, and stress means across sex-specific obesity phenotypes. The mean of DASS components is adjusted for age, marital status, education level, job status, and physical activity level

Table 2 illustrates the distribution of depression, anxiety, and stress levels among phenotypes for men and women. In total, women were more likely to experience higher levels of depression (30.5%), anxiety (44.2%), and stress (43.5%), and the number of severe depression, anxiety, and stress in women (7.3, 15.5, and 16.6%) was higher than men (4.7, 8.2, and 9.5%). The highest and lowest frequency in the entire sample were MHNO women with normal depression levels and MHO men with moderate and severe depression, respectively. The mean scores of depression, anxiety, and stress were compared among different obesity phenotypes after adjusting for age, marital status, level of education, job status, smoking status, and level of physical activity and were illustrated in (Fig. 1). The results showed that mean anxiety scores in men and mean anxiety and stress scores in women were significantly different among obesity phenotypes (*p* = 0.044, *p* = 0.02, and *p* = 0.022, respectively). However, there was no significant difference in the depression scale in both sexes.

**Table 2:** Sex-specific levels of depression, anxiety, and stress in different Obesity phenotypes (*n* = 2469)

	Variables	Total	Metabolically healthy		Metabolically unhealthy		***P***-value
			Non-obese	Obese	Non-obese	Obese	
**Men**	**Depression**						0.051
	Normal	925(79.9)	413 (80.7)	66 (76.7)	282 (79.4)	164 (80.0)	
	Mild	89 (7.7)	27 (5.3)	8 (9.3)	40 (11.3)	14 (6.8)	
	Moderate	90 (7.8)	43 (8.4)	6 (7.0)	24 (6.8)	17 (8.3)	
	Severe	54 (4.7)	29 (5.7)	6 (7.0)	9 (2.5)	10 (4.9)	
	**Anxiety**						0.123
	Normal	810 (69.9)	379 (74.0)	61 (70.9)	239 (67.3)	131 (63.9)	
	Mild	106 (9.2)	40 (7.8)	6 (7.0)	37 (10.4)	23 (11.2)	
	Moderate	147 (12.7)	58 (11.3)	12 (14.0)	52 (14.6)	25 (12.2)	
	Severe	95 (8.2)	35 (6.8)	7 (8.1)	27 (7.6)	26 (12.7)	
	**Stress**						0.493
	Normal	774 (66.8)	353 (68.9)	54 (62.8)	236 (66.5)	131 (63.9)	
	Mild	129 (11.1)	45 (8.8)	13 (15.1)	47 (13.2)	24 (11.7)	
	Moderate	145 (12.5)	65 (12.7)	9 (10.5)	40 (11.3)	31 (15.1)	
	Severe	110 (9.5)	49 (9.6)	10 (11.6)	32 (9.0)	19 (9.3)	
**Women**	**Depression**						0.301
	Normal	911 (69.5)	443 (71.3)	103 (68.2)	163 (71.5)	202 (65.0)	
	Mild	142 (10.8)	64 (10.3)	21 (13.9)	25 (11.0)	32 (10.3)	
	Moderate	162 (12.4)	72 (11.6)	18 (11.9)	28 (12.3)	44 (14.1)	
	Severe	96 (7.3)	42 (6.8)	9 (6.0)	12 (5.3)	33 (10.6)	
	**Anxiety**						0.003
	Normal	732 (55.8)	369 (59.4)	86 (57.0)	128 (56.1)	149 (47.9)	
	Mild	113 (8.6)	48 (7.7)	13 (8.6)	23 (10.1)	29 (9.3)	
	Moderate	263 (20.1)	128 (20.6)	26 (17.2)	48 (21.1)	61 (19.6)	
	Severe	203 (15.5)	76 (12.2)	26 (17.2)	29 (12.7)	72 (23.2)	
	**Stress**						0.472
	Normal	741 (56.5)	362 (58.3)	79 (52.3)	139 (61.0)	161 (51.8)	
	Mild	167 (12.7)	71 (11.4)	25 (16.6)	25 (11.0)	46 (14.8)	
	Moderate	186 (14.2)	87 (14.0)	20 (13.2)	30 (13.2)	49 (15.8)	
	Severe	217 (16.6)	101 (16.3)	27 (17.9)	34 (14.9)	55 (17.7)	

Data are presented as frequency (%)

Table 3 shows the odds ratios (95% CI) of reporting higher levels of depression, anxiety, and stress for different obesity phenotypes for men and women separately. After adjusting for potential confounders, including age, marital status, level of education, job status, smoking status, and level of physical activity, the odds of experiencing higher levels of anxiety were significantly greater in MUO (OR: 1.78, 95% CI: 1.25, 2.54; *p = < 0.001*) and MUNO men (OR: 1.61, 95% CI: 1.17, 2.21; *p = < 0.001*) compared to MHNO men, and also in MUO women (OR: 1.73, 95% CI: 1.28, 2.34; *p = < 0.001*) compared to MHNO women. Moreover, the odds of experiencing higher stress levels were significantly greater in MUNO men (OR: 1.40, 95% CI: 1.02, 1.90; *p = 0.04*) and in MUO women (OR: 1.45, 95% CI: 1.07, 1.96; *p = 0.02*) compared to MHNO men and women, respectively. The difference in having higher depression levels was observed in MUO women before adjustment (OR: 1.39, 95% CI: 1.04, 1.84; *p = 0.02*), but no difference was observed after adjustments in both sexes.

**Table 3:** Odds ratios and 95% confidence intervals for emotional states of different obesity phenotypes (*n* = 2469)

	Models	Status	Depression		Anxiety		Stress	
			OR(95%CI)	***P***-value	OR(95%CI)	***P***-value	OR(95%CI)	***P***-value
**Men**	**Model 1**^**a**^	MHNO	(Ref.)		(Ref.)		(Ref.)	
		MUNO	1.01 (0.72–1.40)	0.97	**1.39 (1.04–1.85)**	**0.03**	1.08 (0.82–1.43)	0.60
		MHO	1.22 (0.71–2.10)	0.46	1.80 (0.73–1.97)	0.48	1.26 (0.79–1.99)	0.33
		MUO	1.08 (0.73–1.61)	0.69	**1.73 (1.24–2.42)**	**< 0.001**	1.32 (0.96–1.83)	0.09
	**Model 2**^**b**^	MHNO	(Ref.)		(Ref.)		(Ref.)	
		MUNO	1.21 (0.79–1.84)	0.31	**1.61 (1.17–2.22)**	**0.003**	**1.40 (1.02–1.90)**	**0.04**
		MHO	1.40 (0.80–2.44)	0.24	1.22 (0.74–2.04)	0.43	1.26 (0.78–2.02)	0.34
		MUO	1.20 (0.79–1.84)	0.39	**1.78 (1.25–2.54)**	**< 0.001**	1.34 (0.95–1.90)	0.09
**Women**	**Model 1**^**a**^	MHNO	(Ref.)		(Ref.)		(Ref.)	
		MUNO	1.01 (0.73–1.40)	0.95	1.14 (0.86–1.52)	0.36	0.91 (0.67–1.22)	0.51
		MHO	1.10 (0.76–1.60)	0.60	1.15 (0.82–1.62)	0.43	1.20 (0.86–1.67)	0.28
		MUO	**1.39 (1.04–1.84)**	**0.02**	**1.65 (1.27–2.13)**	**< 0.001**	1.21 (0.94–1.57)	0.14
	**Model 2**^**b**^	MHNO	(Ref.)		(Ref.)		(Ref.)	
		MUNO	0.80 (0.56–1.16)	0.24	1.16 (0.84–1.61)	0.36	1.04 (0.75–1.46)	0.80
		MHO	0.94 (0.64–1.40)	0.77	1.13 (0.80–1.61)	0.50	1.26 (0.89–1.78)	0.20
		MUO	1.10 (0.78–1.53)	0.60	**1.73 (1.28–2.34)**	**< 0.001**	**1.45 (1.07–1.96)**	**0.02**

a = Unadjusted; b = Adjusted for age, marital status (ref = married), level of education (ref = higher), job status (ref = employed) and level of physical activity (ref = high)

## Discussion

The present study was one of the first attempts to investigate the relation of obesity phenotypes with emotional distress among men and women in Tehran. In total, the current results indicated that women across all phenotypes were more likely to experience depression, anxiety, and stress symptoms compared to men. More stratified analysis based on weight and metabolic status revealed an increased risk of anxiety and stress among MUO women compared to their MHNO counterparts. However, corresponding results for men showed that regardless of weight status, metabolic conditions were associated with higher anxiety and stress levels. Interestingly, obesity phenotypes were not related to depression in either sex.

The current results indicated that women were more likely to report experiencing negative mental health symptoms, mainly in the form of anxiety and stress. Consistent with our findings, sex differences and higher prevalence of mood and anxiety disorders among women have been addressed in Iran [10] and other nations as well [38]. Apart from genetic and other biological factors like different hormonal fluctuations in women [39], some essential psychosocial determinants, including more extended rumination and brooding [40], shame, interpersonal stressors, and experienced violence in women, are considered the reasons for this higher prevalence worldwide. Also, gender inequality, traditional gender roles, and sex-based discrimination are recognized as influential cultural factors [41]. In transitional societies, including most Middle-Eastern countries, all developments in the community’s economic and educational structure in recent decades have been accompanied by the multiplicity of women’s expected roles, which could complicate the underlying causes of mentioned mental health outcomes [42].

There were significant relationships among obesity phenotypes with anxiety and stress in both sexes in the current study. The previous studies focused mainly on depression as a mental health construct regarding obesity phenotypes. On the other hand, using different measurement tools for assessing mental health outcomes, investigating separate effects of obesity and cardiovascular risk factors, and various cultural contexts make the comparison difficult. To the best of our knowledge, only one study among a middle-aged Irish population revealed a higher risk of anxiety in MUO individuals than their MHNO counterparts [26]. Other studies focused on weight and metabolic health status separately. In this regard, a meta-analysis reported a more frequent incidence of anxiety among individuals with obesity compared to those without obesity [12]. Conversely, some evidence showed the lack of association between MetS and anxiety among Japanese men [19]. Regarding stress, the findings of a recent meta-analysis on four studies indicated no association between MetS and stress [24]. In the current study, higher levels of anxiety and stress were simultaneously observed in the same definite phenotypes in both sexes. This is in line with previous findings indicating chronic stress leads to anxiety, and anxiety makes individuals vulnerable to stress [43]. Moreover, indisputable effects of local culture on these global experiences can be seen in the findings; collectivism, one of the fundamental characteristics of Middle Eastern societies, helps prevent depression by social support but may result in increased anxiety. People in high collectivist cultures tend to attribute great significance to the social context, making them more exposed to anxiety [44]. In terms of sex differences, our findings indicated the importance of metabolic health status in increasing men’s anxiety and stress levels, while neither obesity nor metabolic syndrome was solely associated with women’s mental health conditions. One of the potential reasons for this sex difference could be the economic responsibility that men have in Iranian families [45], which could highlight the significance of men’s health conditions.

The current findings revealed no significant association between obesity phenotypes and depression in both sexes. These results are consistent with the English Longitudinal Study of Ageing (ELSA) findings, which indicated that neither obesity nor poor metabolic status was associated with higher risks of depressive symptoms at over 2 years follow-up [28]. Accordingly, another longitudinal survey suggested that obesity was not a predictor of depression in Canadian women [13], and the same results have been observed among Mexican men [15]. Also, the lack of relationship between MetS and depression has been published among a sample of Turkish adults [20]. However, two systematic reviews confirmed the positive relationship between obesity and depression in American and Korean populations [11] and an increased risk of depression for MUO individuals [46]. Since the relation of obesity phenotypes with depression is multifactorial, the discrepancy in the outcomes could have a wide range of physical to psychosocial factors. Aside from gene-by-environment interaction [47], the underlying psychological factors seem to play a particular role in this regard. The increased prevalence of psychiatric morbidity, especially the depression in the treatment-seeking population, somehow reflects the difference in people’s view of obesity [48]. Individuals with obesity who seek possible ways of losing their excessive weight experience obesity as a condition that needs to be changed. This point of view could be caused by psychological factors like perceived body weight [49] and body dissatisfaction [50], which are strongly influenced by western media exposure and negative beliefs about obesity [51]. On the other hand, in some developing countries, obesity is considered a sign of health and wealth.; hence, since higher socio-economic groups are more likely to be obese, excessive weight does not have a significant psychological burden in these communities [51]. Additionally, Muslim countries like Iran have particular dress coding and clothing rules, which may lessen the importance of body shape and appearance and consequently lower psychological effects of weight status [14]. All these factors could make obesity a neutral factor in association with depression levels of the current study’s population.

While the current study had strengths, there were some limitations. To the best of our knowledge, this is the first study to examine the synergic effects of weight and metabolic status on depression, anxiety, and stress among a large population of Tehranian adults. Nevertheless, due to the limitation of the cross-sectional design of the study, the causal relationship could not be established. It was also impracticable to adjust all the confounders due to the complicated essence of emotional distress; thus, the unmeasured variables could affect our findings. Additionally, these results can only be generalized to the Tehranian urban population.

## Conclusions

In conclusion, men and women with various obesity phenotypes experience different anxiety and stress levels. While MUO women and all metabolically unhealthy men experienced more anxiety and stress levels than MHNO individuals, none of the obesity phenotypes were associated with depression. These valuable results on the psychological outcomes associated with different obesity phenotypes would be beneficial to recognize one of the somatic factors contributing to increased anxiety and stress symptoms in adults. These findings could draw the attention of physicians active in the field of metabolic disorders to assess patients’ levels of anxiety and stress and require psychologists to determine the metabolic status of patients with high levels of anxiety and stress. Current results could also improve communities’ public health by planning new strategies specific to each obesity phenotype.

## Supplementary Information

**Additional file 1.**

## Data Availability

The datasets used and/or analyzed during the current study are available from the corresponding author on reasonable request.

## Abbreviations

TLGS: Tehran lipid and glucose study
BMI: Body mass index
MetS: Metabolic syndrome
MHNO: Metabolically healthy non-obese
MHO: Metabolically healthy obese
MUNO: Metabolically unhealthy non-obese
MUO: Metabolically unhealthy obese (MUO)
DASS_21: Depression, anxiety, and stress scale-21 items
