# Supplementary material for: Emotional states of different obesity phenotypes: a sex-specific study in a west-Asian population
Source: BMC Psychiatry. 2021 Mar 4;21:124. doi: 10.1186/s12888-021-03131-3 (PMC7934552; doi:10.1186/s12888-021-03131-3)
Supplement: Supplementary file 1 — Additional file 1. [file 12888_2021_3131_MOESM1_ESM.docx]

**Table 1-Appendix.** Descriptive statistics of BMI and MetS components in men and women

| **Variables** | **Total**  **n=2469** | **Men**  **n=1158** | **Women**  **n=1311** | **P-value**** |
| --- | --- | --- | --- | --- |
| **BMI (Kg/m^2^)** | 27.79±4.85* | 27.4±4.4 | 28.1±5.2 | <0.001 |
| **Waist circumference (cm)** | 93.90±12.06 | 95.6±11.5 | 92.4±12.4 | <0.001 |
| **FBS (mg/dl)** | 97.71±26.72 | 99.5±27.8 | 96.1±25.6 | <0.001 |
| **2 hour blood sugar** | 108 (91-131) | 107 (90-132.25) | 109 (93-130) | 0.30 |
| **Total cholesterol (mg/dl)** | 186.21±39.21 | 183.9±38.5 | 188.2±39.7 | 0.023 |
| **HDL (mg/dl)** | 47.29±11.33 | 42.9±9.7 | 51.1±11.2 | <0.001 |
| **TG (mg/dl)** | 122 (86-173) | 133.5 (94-190) | 112 (80-157) | <0.001 |
| **SBP (mm Hg)** | 114.51±16.69 | 118.6±15.7 | 110.9±16.7 | <0.001 |
| **DBP (mm Hg)** | 76±9.88 | 78.5±9.9 | 73.8±9.3 | <0.001 |

*Mean±SD; BMI: Body mass index; FBS: fasting blood sugar; HDL: High-density lipoprotein; TG: Triglycerides; SBP: Systolic blood pressure; DBP: Diastolic blood pressure.

**P-value refers to the difference between males and females
